# Analyzing Successful Aging and Longevity: Risk Factors and Health Promoters in 2020 Older Adults

**DOI:** 10.3390/ijerph19138178

**Published:** 2022-07-04

**Authors:** Daria A. Kashtanova, Anastasiia N. Taraskina, Veronika V. Erema, Anna A. Akopyan, Mikhail V. Ivanov, Irina D. Strazhesko, Alexandra I. Akinshina, Vladimir S. Yudin, Valentin V. Makarov, Sergey A. Kraevoy, Denis E. Korolev, Irina V. Tarasova, Olga A. Beloshevskaya, Elen A. Mkhitaryan, Olga N. Tkacheva, Sergey M. Yudin

**Affiliations:** 1 Federal State Budgetary Institution “Centre for Strategic Planning and Management of Biomedical Health Risks” of the Federal Medical Biological Agency 10 Bld., 1 Pogodinskaya Str., Moscow 119121, Russia; senreiho@gmail.com (A.N.T.); dveronika784@gmail.com (V.V.E.); mivanov@cspfmba.ru (M.V.I.); akinshina@cspfmba.ru (A.I.A.); vyudin@cspfmba.ru (V.S.Y.); makarov@cspfmba.ru (V.V.M.); skraevoy@cspmz.ru (S.A.K.); yudin@cspmz.ru (S.M.Y.); 2 Russian Clinical Research Center for Gerontology, Pirogov Russian National Research Medical University of the Ministry of Healthcare of the Russian Federation, Bld. 16, 1st Leonova Street, Moscow 129226, Russia; a.alexandrova18@gmail.com (A.A.A.); istrazhesko@gmail.com (I.D.S.); dekorolev@gmail.com (D.E.K.); irish.ff@mail.ru (I.V.T.); olgabell8@mail.ru (O.A.B.); melen99@mail.ru (E.A.M.); tkacheva@rgnkc.ru (O.N.T.)

**Keywords:** longevity, aging, dependence, older adults, dementia, long-lived individuals, geriatric syndromes

## Abstract

Geriatric syndromes (GSs) and aging-associated diseases (AADs) are common side effects of aging. They are affecting the lives of millions of older adults and placing immense pressure on healthcare systems and economies worldwide. It is imperative to study the factors causing these conditions and develop a holistic framework for their management. The so-called long-lived individuals—people over the age of 90 who managed to retain much of their health and functionality—could be holding the key to understanding these factors and their health implications. We analyzed the health status and lifestyle of the long-lived individuals and identified risk factors for GSs. Family history greatly contributes to the health and prevention of cognitive decline in older adults. Lifestyle and certain socioeconomic factors such as education, the age of starting to work and retiring, job type and income level, physical activity, and hobby were also associated with certain GSs. Moreover, the levels of total protein, albumin, alpha-1 globulins, high-density lipoprotein, free triiodothyronine, and 25-hydroxyvitamin D were direct indicators of the current health status. The proposed mathematical model allows the prediction of successful aging based on family history, social and economic factors, and life-long physical activity (f1 score = 0.72, AUC = 0.68, precision = 0.83 and recall = 0.64).

## 1. Introduction

Population aging refers to a rise in the number of people over the working age. The World Health Organization projects that by 2050, more than two billion people worldwide will be over the age of 60 [1]. Russia has also been affected by the aging trend. The distribution of older persons throughout the country, however, is nonuniform: European Russia and Western Siberia have the largest proportion of older adults, while the North Caucasus has the smallest [2]. Population aging, despite its undeniably positive aspects, affects all areas of life, particularly the socioeconomic and healthcare dimensions. This population group is one of the most vulnerable members of society, largely due to aging-associated diseases (ADDs) and geriatric syndromes (GSs). As unequivocally demonstrated by the COVID-19 pandemic, people over 65 are at the highest health risk: the novel virus is known to have affected them in the most severe and deadly way [3,4]. A study of 178,568 COVID-19-related deaths showed that the mortality rate in people over 65 was more than 62 times higher than in people under 55 [5]. The Federal Service for Surveillance on Consumer Rights Protection and Human Wellbeing (Rospotrebnadzor) reported that the risk of COVID-19-related death was 11 times higher in people over 60.

Until recently, loss of functionality, or independence, and other health issues were believed to be inevitable companions of aging. However, as the number of people of an advanced age has grown worldwide, the so-called phenomenon of healthy longevity has gained more and more attention. Healthy longevity is a successful aging, whereby people well over the age of 90, also referred to as long-living people, demonstrate high physical, psychological and social functioning, and delayed onset of AADs and GSs. This phenomenon could hold the key to understanding age-associated risk factors and developing timely prevention strategies aimed at countering senescence and improving the quality of life.

A number of studies have identified factors that could promote successful aging, including a healthy diet and moderate food intake, regular physical activity, and a socially active lifestyle [6], environmental conditions [7], etc. For **instance**, far more people live well into their old age in the so-called blue zones (e.g., Okinawa, Sardinia, and Costa Rica) [7,8,9]. The 20-year follow-up PAQUID (or Paquid) cohort study of people over the age of 70 explored the likelihood of reaching the age of 90 years. The study revealed that the factors affecting the likelihood differed in men and women: physically active men had a higher chance of survival; physically active and relatively healthy women satisfied with their income and housing arrangements had a higher chance of becoming nonagenarians [10]. Genetics is another significant factor of longevity. Brandts et al. found a positive correlation between the paternal lifespan and the life expectancy of male offspring; and the maternal lifespan and the life expectancy of female offspring [11].

In Russia, there has been no comprehensive study on long-living individuals. The Federal State Statistics Service (Rosstat) reports that there are over 30 million people aged 60 and above in Russia [12]. A survey by NAFI Research Centre with 1600 respondents from 50 regions of Russia aged 60 and above [13] showed that 27% (above 7 million) of them live alone and lead socially isolated lives; 30 percent of Russians have a family member who needs constant geriatric care [14], which they often cannot afford; 45% of older adults need support in their daily activities [13].

The present study was carried out in Moscow; therefore, its results cannot be extrapolated to the entire Russian Federation. For instance, the average life expectancy in Moscow is 75 years (71 in men; 78 in women), while the average life expectancy in Russia is 67 years [15]. Recruiting participants in Moscow and the Moscow region was enabled by the proximity of research centers and the availability of geriatric services, social support and healthcare that in recent years have become more accessible to older adults. However, there is still scope for improvement to meet their needs.

Reversing the damage caused by AADs and GSs is more often than not an impossible task. Therefore, effective prevention is crucial. The present study presents an integrated approach to exploring the phenomenon of longevity that included factoring in detailed medical history, risk factor assessment, a comprehensive scale- and survey-based geriatric assessment, and blood testing; the blood test results have been biobanked for further research. This is the first large-scale study on longevity in Russia that made it possible to determine the prevalence of GSs and AADs in older adults (hereinafter, used to refer to people over the age of 90, or long-living individuals/adults) and establish the relationships between various factors and healthy longevity.

## 2. Materials and Methods

### 2.1. Study Design

**Figure 1:**
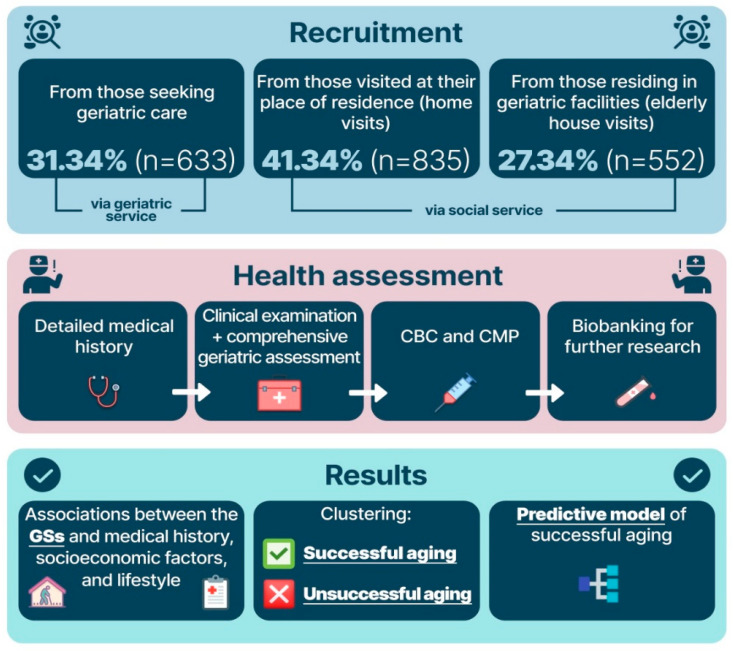
Participant recruitment and study design. Note: CBC—complete blood count; CMP—comprehensive metabolic panel; GSs—geriatric syndromes.

We studied 2020 participants with a verified age of at least 90 years from Moscow and the Moscow region. All participants signed an informed consent form for blood and biomaterial collection and at least two follow-up visits by a physician. The Local Ethics Committee of the Russian Gerontological Research and Clinical Center approved the study (Protocol #30, 24 December 2019). The participant recruitment was carried out with the assistance of social and geriatric services of Moscow and the Moscow region. The recruitment process is presented in Figure 1.

### 2.2. Study Procedures

We analyzed participants’ medical history, socioeconomic and health status, lifetime risk factors for chronic disease and current; factored in all medications taken by the participants; conducted a comprehensive geriatric assessment and screening for 15 GSs following the clinical guidelines of the Russian Ministry of Health [16]:

- Frailty syndrome, diagnosed using the Short Physical Performance Battery;
- Cognitive impairment, assessed by the Mini-Mental State Examination (MMSE) and clock-drawing test;
- Frontal lobe dysfunction, assessed using the Frontal Assessment Battery (FAB) test;
- Chronic pain, assessed based on the questionnaire presented in the Supplementary Materials, Table S1;
- Anxiety disorder, assessed based on the questionnaire presented in the Supplementary Materials, Table S1;
- Risk of falls, assessed based on the questionnaire presented in the Supplementary Materials, Table S1;
- Sensory deficit, assessed based on the questionnaire presented in the Supplementary Materials, Table S1;
- Depression, diagnosed using the Five-Item Geriatric Depression Scale (GDS-5);
- Sarcopenia, diagnosed using the SARC-F questionnaire (strength, assistance walking, rise from a chair, climb stairs, and falls) and hand-held dynamometry;
- Risk of malnutrition, assessed based on the Mini Nutritional Assessment (MNA-scale);
- Fecal or urinary incontinence, self-reported;
- Dependence in ADL (activity of daily living), assessed based on the Barthel index;
- Dependence in IADL (instrumental activity of daily living), assessed based on the Lawton scale;
- Polypragmasia, defined as a simultaneous administration of 5 or more medications;
- Orthostatic hypotension, measured using the standard diagnostic procedure [17].

**Figure 2:**
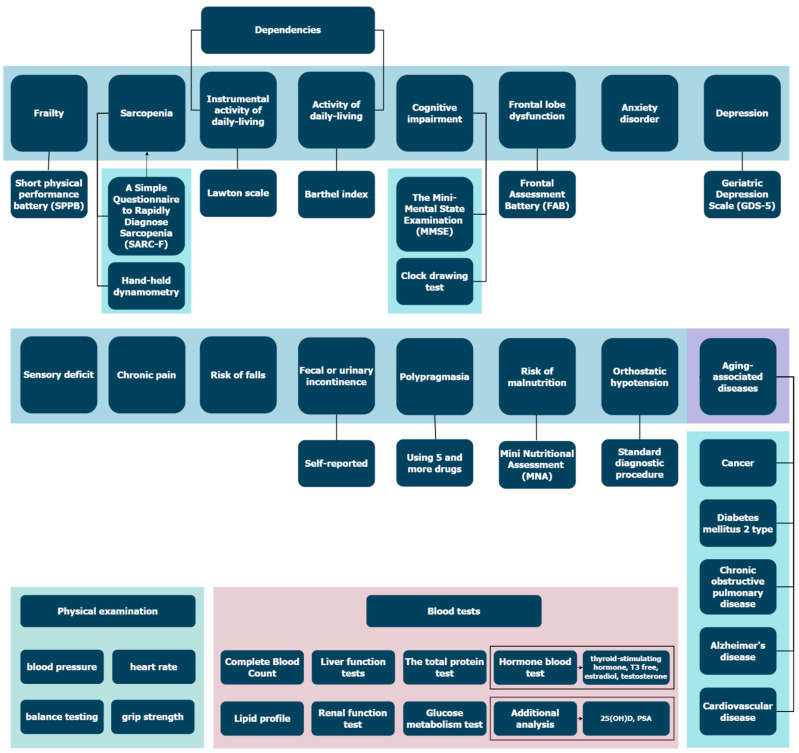
All procedures performed in the study. Please find questionnaires for Sensory deficit, chronic pain, risk of falls, anxiety disorder in Supplementary Materials.

Figure 2 shows all the procedures performed in this study.

## 3. Data Analysis

We used an ordinary least squares Linear Regression Model (Python 3.8 package. Statsmodels) with age and sex as covariates to establish associations between the GSs and factors.

Data modeling function:

y = β_1_ x_1_ + β_2_ x_2_ + β_3_ x_3_ +β_0_

where y is GSs (1 for presence, 0 for absence); x_1_, x_2_, and x_3_ are sex, age, and factors, respectively.

To obtain β we minimized the sum of square differences of the observed and predicted syndrome values, F-tested the significance of the independent variables, and calculated the *p*-value.

To avoid multiple testing problems, we applied the Bonferroni correction.

The Agglomerative Clustering algorithm from the Python 3.8 package scikit-learn 0.24.2 was used for clustering.

To measure the probability of longevity, classification was performed using Logistic Regression (Python 3.8 package. Statsmodels v0.12.2, BSD license, Chapel Hill, NC, USA) with:

$$p=1/\left(1+e^{-\left(\beta_{1}^{}x_{1}+\beta_{2}^{}x_{2}+\ldots +\beta_{n}^{}x_{n}+\beta_{0}\right)}\right)$$

where x is the predictors, β the coefficient found by the trained model.

## 4. Results

### 4.1. Clinical Characteristics of the Participants

A total of 2020 participants were screened: 504 men (25%) and 1516 women (75%) of the average age of 93 ± 2.5 years old (with a maximum of 107 and a minimum of 90 years old). The long-living men were more physically active than the long-living women. On average, all participants had 1 ± 1 AADs with a higher prevalence of certain AADs in men (cancer and CVD) and received 5 ± 2 medications. The most common GSs were sensory deficits and dependence in ADL and IADL. Moreover, many GSs were more common in women. Overall, women had more GSs (mean = 9.74) than men (mean = 8.61) (*p*-value < 0.002, significance for all baseline characteristics (Table 1).

### 4.2. Associations and Correlations between the GSs and Evaluated Factors

We analyzed the relationships between successful aging, healthy longevity, lifestyle, and medical history. To identify predictors of healthy longevity, we grouped the factors (Supplementary Materials, Table S2) as follows:

- Family history;
- Socioeconomic factors;
- Lifestyle and physical activity;
- Obstetrics and gynecological history;
- Factors reflecting current health status.

We used a linear regression model to determine correlations and associations between GSs and evaluated factors. The Bonferroni correction was applied to the *p*-value threshold.

#### 4.2.1. Assessment of the Associations between the GSs and Factors

**Figure 3:**
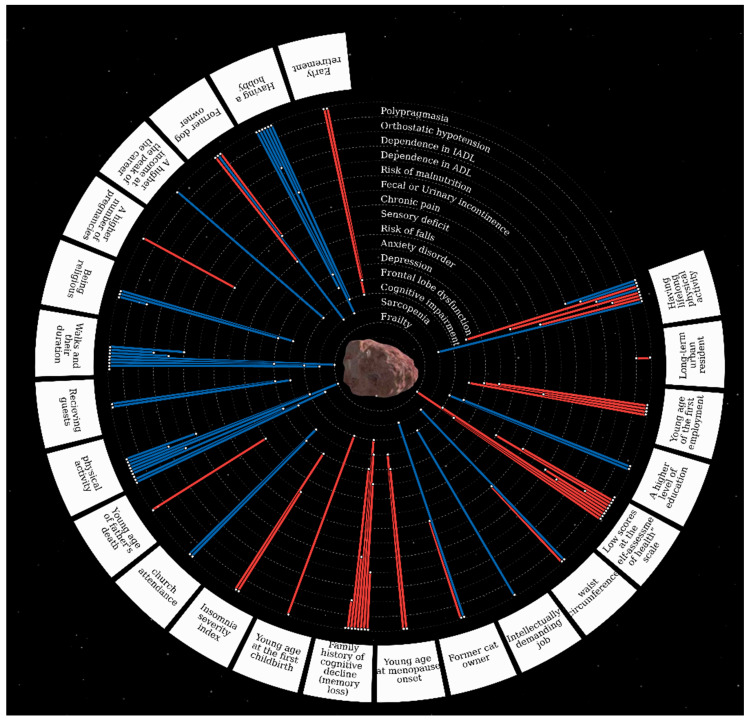
The associations between the GSs and evaluated factors.* The centerpiece Ananke (from Ancient Greek Ἀνάγκη for inevitability, fate, adversity, and necessity) is Jupiter’s satellite, also known as Jupiter XII. Geriatric syndromes are located in orbits around the satellite. Risk factors for geriatric syndromes are located along the outer contour. The red line means the direct relationship between the risk factor and GSs, the blue line means the inverse relationship (protective factors).

Figure 3 and Table 2 show statistically significant associations between the GSs and the analyzed factors (the detailed table of the associations between the GSs and analyzed factors is presented in the Supplementary Tables S3–S5).

We found that family history had a profound correlation with successful aging. For instance, father’s death at a young age was strongly associated with the risk of falls and frontal lobe dysfunction; family history of cognitive decline was correlated with almost all GSs, such as incontinence, dependence in ADL, sarcopenia, depression, risk of malnutrition, cognitive impairment, frontal lobe dysfunction, polypragmasia, AADs, orthostatic hypotension, and anxiety.

Many GSs were also associated with socioeconomic factors and lifestyle: working from a young age was associated with frontal lobe dysfunction, chronic pain, depression, and anxiety; early retirement was correlated with a higher prevalence of frontal lobe dysfunction and cognitive impairment; higher levels of education, intellectually demanding jobs, and higher income were associated with a lower prevalence of frontal lobe dysfunction and cognitive impairment.

Having a hobby was inversely correlated with the prevalence of frontal lobe dysfunction and cognitive impairment, depression, sarcopenia, risk of malnutrition, and dependence in IADL. Having a pet contributed to a lower risk of sarcopenia. Participants who were more likely to have guests over were at a lower risk of frontal lobe dysfunction and depression. Depression, frontal lobe dysfunction, and orthostatic hypotension were less common in religious participants, but religion was associated with risk of malnutrition. Polypragmasia was more common in the participants who lived in urban areas.

Lifelong physical activity was correlated with a lower prevalence of sarcopenia, dependence ADL and IADL, and the risk of malnutrition. Physical activity was positively correlated with a higher risk of falls, orthostatic hypotension, and frontal lobe dysfunction. However, it should be noted that physical activity implies both physical activities in a broad sense and hard labor. These correlations were significant only in participants who had engaged mainly in heavy physical labor (*p*-value = 9.57 × 10^−9^ for orthostatic hypotension and *p*-value = 1.59 × 10^−10^ for frontal lobe dysfunction). Physical activity was associated with a lower prevalence of cognitive impairment in participants who had engaged in intellectually demanding activities (*p*-value = 4.98 × 10^−5^).

It is hardly surprising that we found associations between several obstetric and gynecological factors and GSs: the first childbirth at an early age was associated with a higher prevalence of cognitive impairment, and a greater number of pregnancies was associated with chronic pain; early onset menopause was associated with a higher risk of frontal lobe dysfunction, depression, and orthostatic hypotension.

Current health status analysis revealed that a larger waist circumference was associated with a lower prevalence of sarcopenia and a higher prevalence of chronic pain. Participants who remained physically active and frequently took walks were less likely to suffer from frontal lobe dysfunction, cognitive impairment, depression and sarcopenia, and were at a lower risk of malnutrition. Insomnia was associated with a lower prevalence of depression and sensory deficits.

#### 4.2.2. Assessment of the Association between the Laboratory Test Results, GSs and Presence of ADDs

The associations between GGs and blood test results were established using a linear regression model. Table 3 shows the significant associations between GSs and blood test results: increased levels of total protein, albumin, free T3, and alpha-1 globulin were correlated with a lower prevalence of a number of GSs.

The most significant association was found between total protein levels and risk of malnutrition; albumin levels and cognitive impairment, ADL and IADL, frailty, sarcopenia, and the risk of malnutrition.

### 4.3. Clustering for Patterns of the Most Successful and Unsuccessful Aging

#### 4.3.1. Successful Aging Cluster

**Figure 4:**
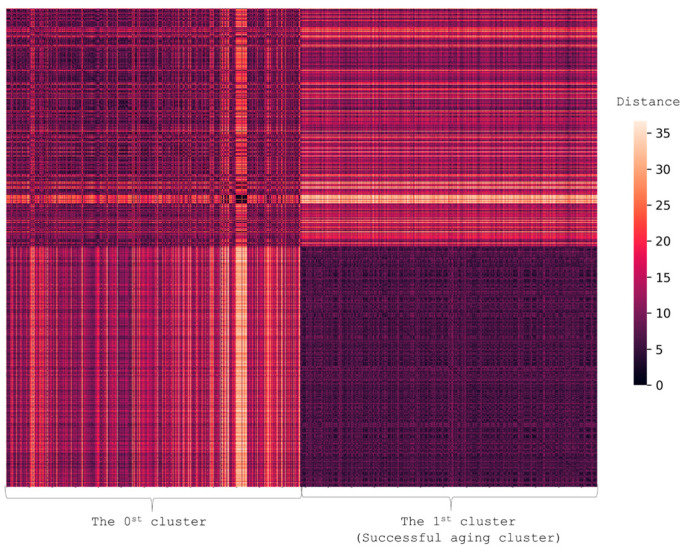
Heatmap illustrating the difference in the presence of AADs and GSs. Each person is represented by a vector with values showing the presence of AADs (cancer, CVD, DM2, and COPD) and scores on MMSE, FAB, and SPPB. The figure shows the pairwise distance matrix for the selected set of individuals in Euclidean space. The X-axis is selected set of individuals and the Y-axis is the same set (the matrix is symmetric). The lighter the pixels, the bigger the difference between the pair in terms of selected features. The heatmap shows that the successful aging cluster (Cluster 1) differed from Cluster 0.

To divide 1696 participants into groups, we performed clusterization based on the presence of AADs (cancer, CVD, DM2, and COPD) and scores on MMSE, FAB, and SPPB. This resulted in two clusters (Figure 4) (*p*-value for difference 9.6 × 10^−6^).

Cluster 1 was a group of 850 healthier individuals of relatively the same age (94 ± 2 years old) and included more men than Cluster 0. Family history analysis showed that the risk of cognitive decline in Cluster 1 was three times lower than in Cluster 0. Cluster 1 participants shared the following socioeconomic characteristics: they had higher levels of education; had started to work on average two years earlier, and had retired on average three years later than their peers; they had more intellectually demanding occupations and earned more. Women in this group experienced the onset of menopause on average three years later than their peers. These participants also demonstrated higher functionality. Physical examination revealed that they had a larger waist circumference, higher weight, and a higher BMI. They performed better on the Clock-drawing and Age is-not-a-barrier tests, and had lower Charlson comorbidity indices. They self-assessed their health and quality of life much higher compared to other participants; led a more socially and physically active lifestyle; had fewer physical limitations; were more religious and had lifelong hobbies.

Other differences are presented in Table 4.

#### 4.3.2. Cluster of the Least Successful Aging

**Figure 5:**
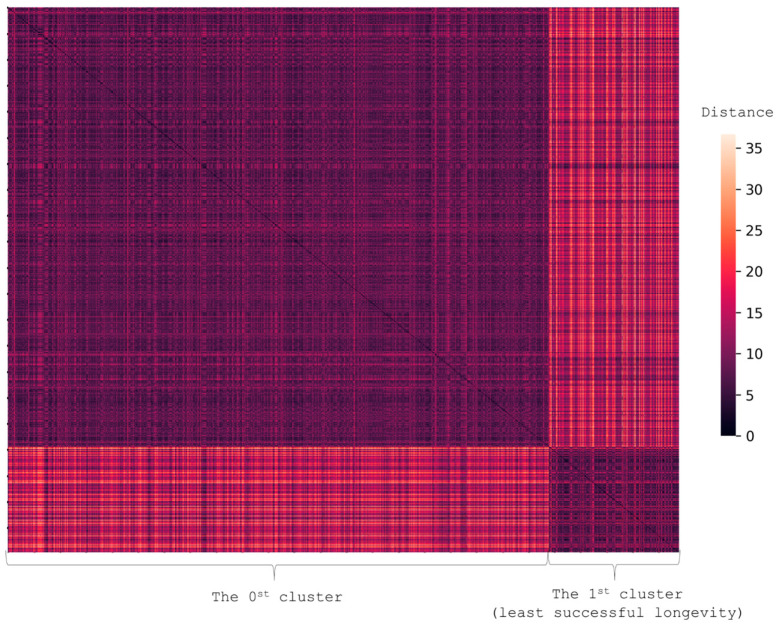
The heatmap shows two clusters — the least successful aging and the other one. Each person is represented by a vector with values showing the presence of AADs (cancer, CVD, DM2, and COPD) and scores on MMSE, FAB, and SPPB. The figure shows the pairwise distance matrix for the selected set of individuals in Euclidean space. The X-axis is selected set of individuals and the Y-axis is the same set (the matrix is symmetric). The lighter the pixels, the bigger the difference between the pair in terms of selected features.

To identify participants with the highest number of health problems, we clustered the least successful aging group based on the following factors: presence of cognitive decline, frailty, sensory deficit, and ADDs. We obtained two clusters (Table 5, Figure 5) (*p*-value for difference 1.1 × 10^−5^).

There were 260 individuals of the average age of 94 ± 3 years old in the unsuccessful aging cluster. They shared the following characteristics: they had lower levels of education; had retired on average 3 years earlier than their peers; had more physically demanding occupations, and earned less. Lifetime hobbies were rare in this group. Physical examination revealed that the participants from the 1st least successful cluster had a smaller waist circumference. They scored lower on the clock-drawing test and GDS-5 and were more frequently affected by hippocampal dysfunction, Alzheimer’s disease, and AADs. They also led less socially and physically active lifestyles and self-assessed their health and quality of life significantly lower than other participants.

### 4.4. Predicting Successful Aging

Based on the clustering described in Section 4.3, we attempted to predict the probability of successful aging based on the long- and medium-term factors using a logistic regression model. Each participant was assigned a number: 1 for the successful aging cluster and 0 for others. We used age and sex (1 for men, 2 for women) as additional factors to the following set:

- Family history of cognitive decline (1—yes; 0—no);
- Physical activity for the most part of one’s life;
- Mother’s age at death;
- Father’s age at death;
- Income at the peak of the career (0—low, 1—medium, 2—high);
- Total number of children;
- Area of residence (1—non-urban, 2—urban);
- Type of job (0—physical work, 2—intellectually demanding work, 1—both);
- Former dog owner (1—true, 0—false);
- Former cat owner (1—true, 0—false);
- Disability (0—none, 1—40–60% of functionality loss, 2—70–80% of functionality loss, 3—90–100% of functionality loss);
- Marital status (1—married or other type of relationship, 2—widow/widower, 3—divorced/separated, 4—never married);
- Age of retirement;
- Age of starting to work;
- Hobby (1—yes, 0—no);
- Education (0—primary school, 1—middle school, 2—high school, 3—high school and vocational training, 4—vocational training, 5—incomplete higher education, 6—complete higher education, 7—doctoral degree);
- Religion (0—non-religious, 1—religious);
- Grip strength;

The model was trained on 694 samples and tested on 174 samples. We obtained f1 score = 0.72 and AUC = 0.68; precision = 0.83 and recall = 0.64. We also factored in the age of menopause onset as a distinguishing factor. The sample size was thus 483 for training and 121 (women only) for testing. The AUC increased to 0.69, the f1-score to 0.73, the precision to 0.84, and the recall remained unchanged. The age of menopause onset showed the highest coefficient (0.7). This model included the following most significant factors: age, sex, hobby, religion, education, age of retirement, physical activity, family history of cognitive decline, and, for women, the age of menopause onset.

## 5. Discussion

The present study was a comprehensive assessment of the unique population group—long-lived men and women. Both in this study and on a larger population scale, long-lived women outnumber long-lived men; however, we analyzed the gender differences and discovered that women suffered from more GSs and were less physically active. This fact confirms the so-called male–female health-survival paradox [18]. Therefore, we can assume that men need to be much healthier and more physically active to achieve longevity. Despite a higher prevalence of GSs in women, AADs (cancer and cardiovascular diseases) were more common in men. In this study, we did not specify the localization of cancer. However, current data show that prostate cancer (that has almost a 100% 5-year survival rate [19]) is the most common disease in men aged 60 and older. This fact could account for the difference in the cancer rates between men and women. CVDs are generally known to be more prevalent in men.

The clusterization and individual association analysis revealed that no dementia in family history greatly contributes to the preservation of cognitive functions, physical activity, and physical fitness in very old adults. Similar results obtained in studies of younger participants support these findings [20,21].

Working from an early age, lower levels of education, and physical labor for most of one’s life were associated with many GSs. Other studies [22,23] support these findings. We also identified factors correlated with a lower prevalence of GSs. On average, the successful aging cluster participants started working 2 years later and retired 3 years later than their peers. Usually, they had engaged in more intellectually demanding occupations and earned more. The impact of socioeconomic factors on cognitive functions and some GSs has been confirmed in several studies [24,25,26,27]. Thus, we can consider higher levels of education, intellectually demanding occupation throughout life, high income, and no family history of cognitive decline as anti-risk factors for healthy longevity. These associations have been previously established in earlier studies; however, their results mostly pertain to younger cohorts.

Socialization was another important promoter of healthy longevity. Those participants who more frequently had guests over and had hobbies suffered from fewer GSs. Owning a cat or dog was associated with stronger and healthier muscles. Remarkably, having religious beliefs was to a certain extent a protective factor associated with healthy longevity. This has previously been confirmed in several studies [28,29].

Physical activity is known to play an important role in the prevention of ADDs. It also lowers the risk of GSs and increases functionality in older adults [30]. A systematic review of almost 8000 participants showed that low levels of physical activity and a sedentary lifestyle were associated with frailty in community-dwelling older adults [31]. Therefore, it is not surprising that in our study we found that life-long physical activity reduced the risk of sarcopenia, dependence in ADL and the number of AADs. Grip strength as an indicator of physical activity was inversely correlated with sarcopenia, frailty, dependence in IADL, and ADDs. Participants in the cluster of successful aging were more physically active and had fewer physical limitations. The results of the comprehensive assessment of the associations between physical activity and GSs are also of great interest: high-level lifelong physical activity was shown to increase the risk of falls in long-lived individuals and to be associated with orthostatic hypotension and chronic pain, but only if it involved high-intensity work.

It is noteworthy that the successful aging cluster participants had higher BMIs and larger waist circumferences, which demonstrates a protective effect of a slightly increased body weight in old adults [32].

We found several risk factors for GSs in women. A higher number of pregnancies was associated with a higher prevalence of chronic pain. We did not find a connection between the number of deliveries and chronic pain, but there was a positive correlation between the assumed number of abortions and increased chronic pain (*p*-value = 3.21 × 10^−6^, coefficient of correlation—0.14). Women who gave birth to their first child when they were under 25 years of age were characterized by a lower level of education (*p*-value = 1.73 × 10^−5^ from *t*-test for difference) and lower income at the peak of their careers (*p*-value = 0.01). Moreover, in this group of participants, cognitive impairment and frontal lobe dysfunction were more common (OR = 1.31, *p*-value = 0.04 and OR = 1.35, *p*-value = 0.04, respectfully). These findings are corroborated by other researchers [33,34]. There was also a link between early onset menopause and frontal lobe dysfunction syndrome and depression. Georgakis M.K. et al. confirmed the connection between the duration of menopause and the prevalence of depression in a systematic review [35]. There was research showing that menopausal hormone therapy (MHT) reduced the risk of dementia and Alzheimer’s disease [36]. Thus, early menopause is a modifiable risk factor and could be reversed by a timely MHT [37].

In blood tests, we were also able to highlight the parameters that had the most significant association with GSs. Reduced levels of total protein were associated with six GSs, and reduced levels of albumin with nine. Meta-analysis of over 50,000 participants showed that total protein and albumin were reliable indicators of malnutrition [38]. Reduced serum albumin levels were associated with aging and mortality in older people [39]. Lower levels of total protein and albumin were linked to sarcopenia [40] and cognitive impairment [41].

In our study, we found that increased levels of alpha-1 globulins were associated with four GSs. The level of alpha-1 globulins had the strongest association with the prevalence of frontal lobe dysfunction. Alpha-1 proteins, i.e., alpha-1 antitrypsin, alpha-1 acid glycoprotein, and transport proteins are acute-phase proteins. Increased levels of alpha-1 proteins and, specifically, alpha-1 antitrypsin and alpha-1 acid glycoprotein, were associated with systemic inflammation [42,43]. These comprise a composite biomarker GlycA that, among other disorders, indicates cognitive impairment [44] and mortality [45]. Inflammation is one of the fundamental abnormal physiological processes associated with aging, frailty [46], and sarcopenia [47]. Inflammation is crucial to the onset and progression of diabetes, cardiovascular diseases, Alzheimer’s disease, and other chronic aging-related diseases [48,49].

In our study, reduced levels of free T3 were associated with five GSs, including the risk of malnutrition, frailty, and sarcopenia. Previously, it was demonstrated that lower free T3 levels were associated with grip strength, SPPB scores [50,51], and the risk of frailty in older adults [52,53], including long-lived individuals [54].

We found that reduced levels of vitamin D were associated with frailty, cognitive impairment, and aging-associated diseases. Cholecalciferol deficiency was associated with molecular mechanisms that underlie aging: cell senescence, inflammation, oxidative stress, and others. Vitamin D levels were linked to functional status, physical performance, and presence and severity of GS and aging-related diseases [55,56].

We found that higher levels of high-density lipoproteins were correlated with a lower prevalence of ADDs, which has been confirmed in other studies on younger participants [57,58]. Reduced high-density lipoprotein levels were also associated with three GSs, including impaired cognitive function, dependence ADL, and frailty. Other studies have also confirmed that increased levels of high-density lipoproteins have an additional protective effect against dependence in ADL [59]. Notably, no association was found between the GSs and low-density lipoproteins.

Another defining feature of the present study is the development of the clusters and a successful aging calculator, based not only on the current health status but also on a wide variety of socioeconomic factors and medical history.

## 6. Conclusions

The present study of long-lived individuals is unprecedented in its scope and thoroughness. It is worth mentioning that all participants consented to biobanking their blood, stool, and saliva samples for further research. As to be expected, some of the findings are based on the participants’ responses and are, therefore, subjective (for example, responses concerning their past lifestyles). This fact constitutes a limitation of the study. Moreover, all participants were from Moscow and the Moscow region.

Successful aging is a complex phenomenon that depends on a variety of medical, socioeconomic, and environmental factors. It can be linked to potentially modifiable habits and lifestyles. Different statistical methods showed that physical and social activity, economic well-being, and higher levels of education could be associated with successful aging. Further research of this phenomenon could help identify modifiable factors that promote successful aging in all groups of the population.

## Figures and Tables

**Table 1:** Baseline characteristics of the participants and their distribution between men and women.

	In All Participants (n = 2020)	In Men (n = 504)	In Women (n = 1516)	*p*-Value for the Difference between Men and Women
age (mean ± sd)	93 ± 2.5	94 ± 2.39	94 ± 2.58	0.61
BMI (mean ± sd)	25.91 ± 4.5	25.45 ± 3.61	26.07 ± 4.76	0.009
**Smoke status n (%)**				
current smoker	9 (0.5%)	6 (1%)	3 (0.2%)	<0.001
ex-smoker	231 (12%)	195 (40%)	36 (2%)	
**Physical activity n (%)**				
Unable to get out of bed **	269 (14%)	37 (7%)	232 (16%)	<0.001
Able to move inside the house but unable to leave the house **	618 (31%)	117 (23%)	501 (34%)	<0.001
Leaves the house only when necessary	262 (13%)	60 (12%)	202 (14%)	0.41
Able to go for a walk *	715 (36%)	231 (46%)	484 (33%)	<0.001
Performs physical exercise *	117 (6%)	53 (11%)	64 (4%)	<0.001
**Presence of AADs**				
Cancer *	141 (7%)	61 (12%)	80 (5%)	<0.001
CVD *	1380 (69%)	369 (74%)	1011(68%)	<0.001
DM2	291 (15%)	59 (12%)	233 (16%)	0.04
Alzheimer’s disease	272 (18%)	53 (14%)	219 (19%)	0.01
COPD	277 (14%)	86 (17%)	191 (13%)	0.01
Average number of AADs (mean ± sd)	1 ± 1	1 ± 1	1 ± 1	0.22
Average number of received medicines (mean ± sd)	5 ± 2	5 ± 2	4 ± 2	0.35
**Presence of GSs**				
Cognitive impairment **	1016 (53%)	207 (43%)	809 (56%)	<0.001
Frontal lobe dysfunction	1495 (77%)	358 (73%)	1137(78%)	0.03
Chronic pain **	1236 (63%)	277 (55%)	959 (65%)	<0.001
Depression **	956 (49%)	205 (42%)	751 (51%)	<0.001
Anxiety disorder **	471 (36%)	96 (27%)	375 (38%)	<0.001
Orthostatic hypotension	361 (27%)	113 (30%)	248 (26%)	0.14
Risk of falls **	1122 (57%)	247 (49%)	875 (59%)	<0.001
Sensory deficit	1841 (94%)	463 (92%)	1378(94%)	0.34
Sarcopenia	1417 (83%)	369 (83%)	1048(82%)	0.63
Fecal or urinary incontinence **	1464 (73%)	309 (61%)	1155(76%)	<0.001
Dependence in ADL **	1800 (92%)	402 (82%)	1398(94%)	<0.001
Dependence in IADL	1859 (94%)	450 (91%)	1409(95%)	0.005
Polypragmasia	935 (51%)	236 (51%)	699 (50%)	0.69
Frailty **	1786 (89%)	411 (81%)	1375(90%)	<0.001
Risk of malnutrition **	1517 (86%)	346 (77%)	1171(89%)	<0.001
Average number of GS (mean ± sd)	10 ± 2	9 ± 2	10 ± 2	<0.001

Note: * more frequently observed in men; ** more frequently observed in women. BMI—body mass index, CVD—cardiovascular diseases, DM2—diabetes mellitus, COPD—chronic obstructive pulmonary disease, AADs—aging-associated diseases, ADL—activity of daily living, IADL—instrumental activity of daily living, GS—geriatric syndrome.

**Table 2:** The statistically significant associations between the GSs and evaluated factors (*p*-value < 0.001).

Factor	GS	CC or OR	**n**
**Family history**			
Father’s age at death (median = 68, IQR = (50, 76))	Risk of falls	CC −0.14	1518
Relative with cognitive decline (memory loss)	Fecal or urinary incontinence	OR 2.1	1784
	Depression	OR 2.85	1737
	Risk of malnutrition	OR 2.91	1611
	Frontal lobe dysfunction	OR 4.47	1725
	Orthostatic hypotension	OR 1.82	1229
	Anxiety disorder	OR 1.81	1266
	Cognitive impairment	OR 2.43	1704
**Socioeconomic factors**			
Education	Cognitive impairment	CC −0.23	1854
	Frontal lobe dysfunction	CC −0.16	1883
Age of the first employment (median = 17, IQR = (14, 20))	Depression	CC −0.13	1723
	Frontal lobe dysfunction	CC −0.29	1716
	Chronic pain	CC −0.13	1763
	Anxiety disorder	CC −0.1	1235
job type	Cognitive impairment	CC −0.18	1827
Age of retiring (median = 64, IQR = (57, 70))	Cognitive impairment	CC −0.14	1649
	Frontal lobe dysfunction	CC −0.13	1680
Income at the peak of the career	Cognitive impairment	CC −0.181	1397
**Obstetric and gynecological history**			
Age at the first childbirth (median = 24, IQR = (22, 27))	Cognitive impairment	CC −0.11	1031
Number of pregnancies (median = 2, IQR = (1, 3))	Chronic pain	CC 0.11	1274
Age at menopause onset (median = 50, IQR = (48, 55))	Orthostatic hypotension	CC −0.17	791
	Depression	CC −0.15	1096
	Frontal lobe dysfunction	CC −0.31	1095
	Dependence in IADL	CC −0.1	1096
**Lifestyle**			
Long-term urban resident	Polypragmasia	OR 2.43	1422
Religion	Orthostatic hypotension	OR 0.57	1305
	Depression	OR 0.62	1852
	Risk of malnutrition	OR 1.07	1716
	Frontal lobe dysfunction	OR 0.43	1848
Church attendance	Frontal lobe dysfunction	CC −0.126	1852
	Depression	CC −0.112	1857
Having a hobby	Cognitive impairment	OR 0.28	1341
	Risk of malnutrition	OR 0.32	1291
	Sarcopenia	OR 0.35	1277
	Dependence in IADL	OR 0.31	1386
	Depression	OR 0.59	1352
	Frontal lobe dysfunction	OR 0.61	1361
Current cat owner	Sarcopenia	OR 0.47	1307
Former cat owner	Chronic pain	OR 2.82	1446
Current dog owner	Sarcopenia	OR 0.49	1310
	Risk of falls	OR 1.79	1459
Former dog owner	Chronic pain	OR 1.87	1451
Receiving guests	Depression	CC −0.15	1851
	Frontal lobe dysfunction	CC −0.138	1842
**Physical activity**			
Lifelong physical activity	Sarcopenia	OR 0.27	1259
	Risk of falls	OR 1.85	1387
	Orthostatic hypotension	OR 2.79	960
	Frontal lobe dysfunction	OR 1.82	1341
	Dependence in ADL	OR 0.36	1351
	Dependence in IADL	OR 0.36	1368
	Chronic pain	OR 2.33	1383
	Risk of malnutrition	OR 0.63	1272
Current physical activity	Cognitive impairment	CC −0.267	1876
	Frailty	CC −0.251	1978
	Dependence in ADL	CC −0.256	1934
	Depression	CC −0.225	1909
	Fecal or urinary incontinence	CC −0.206	1977
	Sarcopenia	CC −0.166	1703
	Frontal lobe dysfunction	CC −0.148	1905
	Risk of malnutrition	CC −0.324	1752
	Dependence in IADL	CC −0.263	1943
Walks and their duration	Risk of malnutrition	CC −0.325	1754
	Dependence in IADL	CC −0.253	1942
	Dependence in ADL	CC −0.248	1932
	Frailty	CC −0.223	1978
	Depression	CC −0.202	1907
	Cognitive impairment	CC −0.21	1877
	Sarcopenia	CC −0.172	1706
	Fecal or urinary incontinence	CC −0.17	1977
**Factors, reflecting current health status**			
Insomnia severity index (median = 8, IQR = (4, 13))	Depression	CC −0.319	251
	Sensory deficit	CC −0.239	246
Waist circumference (median = 89, IQR = (80, 97))	Chronic pain	CC −0.125	1643
	Sarcopenia	CC −0.102	1518
Health self-assessment	Frontal lobe dysfunction	CC −0.312	1675
	Frailty	CC −0.22	1711
	Cognitive impairment	CC −0.198	1637
	Dependence in ADL	CC −0.194	1674
	Risk of malnutrition	CC −0.198	1571
	Orthostatic hypotension	CC −0.192	1238
	Risk of falls	CC −0.163	1711
	Chronic pain	CC −0.16	1708
	Fecal or urinary incontinence	CC −0.124	1709

Note: OR—odds ratio; CC—correlation coefficient; IQR—interquartile range. ADL—activity of daily living, IADL—instrumental activity of daily living, GS—geriatric syndrome.

**Table 3:** Assessment of the association between the laboratory test results, GSs and presence of ADDs (only significant, *p*-value < 0.001).

Blood Test Results	GSs	CC	n
Total protein (median = 70, IQR = (65, 75))	Dependence in IADL	CC −0.09	1937
	Frailty	CC −0.08	1978
	Frontal lobe dysfunction	CC 0.14	1893
	Risk of malnutrition	CC −0.13	1735
	Sarcopenia	CC −0.09	1691
	Orthostatic hypotension	CC −0.14	1296
Alpha 1 globulins (median = 3.2, IQR = (2.9, 3.5))	Cognitive impairment	CC 0.08	1862
	Frailty	CC 0.08	1974
	Frontal lobe dysfunction	CC 0.13	1891
	Risk of malnutrition	CC 0.12	1734
Cholesterol HDL (median = 1.26, IQR = (1.06, 1.53))	Cognitive impairment	CC −0.09	1874
	Dependence in ADL	CC −0.07	1937
	Frailty	CC −0.08	1987
T3 free (median = 3.6, IQR = (3.2, 4))	Cognitive impairment	CC −0.08	1868
	Dependence in IADL	CC −0.07	1938
	Frailty	CC −0.1	1980
	Risk of malnutrition	CC −0.14	1736
	Sarcopenia	CC −0.1	1692
25-OH-D (median = 8, IQR = (5, 12))	Cognitive impairment	CC −0.13	1872
	Dependence in ADL	CC −0.1	1934
	Dependence in IADL	CC −0.1	1944
	Frailty	CC −0.1	1985
	Polypragmasia	CC 0.09	1802
	Risk of malnutrition	CC −0.11	1743
Albumin (median = 35, IQR = (38, 41))	Chronic pain	CC 0.08	1921
	Cognitive impairment	CC −0.15	1862
	Dependence in ADL	CC −0.12	1924
	Dependence in IADL	CC −0.13	1933
	Frailty	CC −0.14	1974
	Incontinence	CC −0.08	1970
	Risk of malnutrition	CC −0.18	1734
	Sarcopenia	CC −0.14	1690
	Orthostatic hypotension	CC 0.09	1295
Total protein (median = 70, IQR = (65, 75))	Presence of AADs	CC −0.13	1975
Cholesterol HDL (median = 1.26, IQR = (1.06, 1.53))		CC −0.06	1984
T3 free (median = 3.6, IQR = (3.2, 4))		CC −0.11	1977
25-OH-D (median = 8, IQR = (5, 12))		CC −0.08	1982
Albumin (median = 28.3, IQR = (35.1, 41.5))		CC −0.14	1971

Note: n—total cohort size for association analysis; IQR—interquartile range; CC—correlation coefficient; HDL—high density lipoproteins; T3—triiodthyronin; AADs—aging-associated diseases; ADL—activity of daily living; IADL—instrumental activity of daily living; GS—geriatric syndrome.

**Table 4:** Differences in factors (only significant) in the successful aging cluster.

		Cluster 0			Cluster 1. Successful Aging			*p*-Value		
men		172			259			2.14 × 10^−6^		
women		674			591					
age (median [IQR]))		94 (92, 96)			93 (92, 95)					
**Family history**										
Family history of cognitive decline		26%			8%			3.95 × 10^−21^		
**Lifestyle**										
Religion		72%			79%			2.47 × 10^−4^		
Life-long hobby		36%			48%			5.88 × 10^−6^		
**Education**										
Primary education		15%			4%			1.34 × 10^−19^		
Middle school		13%			12%					
High school		21%			15%					
Vocational education		5%			5%					
High school and vocational education		16%			16%					
Incomplete higher education		1%			2%					
Higher education		27%			41%					
Doctoral degree		2%			5%					
**Job type**										
Intellectually and physically demanding job		33%			18%			3.76 × 10^−11^		
Intellectually demanding job		29%			38%					
Physically demanding job		38%			44%					
Age of the first employment		17 (14, 20)			19 (15, 20)			9.43 × 10^−15^		
Age of retiring		60 (55, 68)			65 (60, 70)			3.55 × 10^−13^		
Currently employed		<1%			2%			7.83 × 10^−5^		
**Income at the peak of the career**										
Low		17%			6%			3.4 × 10^−6^		
Middle		71%			79%					
High		13%			15%					
**Disability**										
no		16%			15%			3.34 × 10^−4^		
40–60% of functionality loss		3%			3%			6.35 × 10^−8^		
70–80% of functionality loss		58%			70%					
90–100% of functionality loss		23%			12%					
**Obstetrics-gynecologic history**										
Age of the menopause onset		50 (48, 52)			52 (50, 55)			1.57 × 10^−18^		
**Physical examination**										
Clock-drawing test		4 (0, 6)			8 (5, 10)			1.22 × 10^−77^		
Weight		65 (56, 74)			69 (60, 78)			3.19 × 10^−8^		
Waist circumference		88 (78, 96)			90 (83, 98)			1.07 × 10^−6^		
MMSE *		18 (13, 22)			26 (24, 28)			1.02 × 10^−194^		
Age is not a barrier test		4 (3, 5)			6 (2, 4)			1.98 × 10^−25^		
SPPB *		1 (0, 3)			5 (2, 7)			2.64 × 10^−92^		
Charlson comorbidity index		2 (1, 4)			1 (0, 3)			9.11 × 10^−31^		
BMI		25 (22.5, 28)			26.1 (23.6, 29.2)			1.98 × 10^−6^		
GDS-5		2 (1, 4)			1 (0, 2)			3.09 × 10^−36^		
**Vision without glasses**										
Good		19%			22%			4.74 × 10^−5^		
Average		34%			43%					
Bad		47%			35%					
**Syndromes and diseases**										
Frontal lobe disorder *		97%			43%			7.72 × 10^−121^		
Hippocampus dysfunction		55%			11%			4.64 × 10^−78^		
Alzheimer’s disease		34%			46%			3.95 × 10^−39^		
Health self-assessment (10 grade scale)		5 (3, 6)			6 (5, 7)			1.49 × 10^−42^		
Quality of life self-assessment (10 grade scale)		5 (4, 7)			7 (6, 8)			8.38 × 10^−31^		
Number of aging-associated diseases		1 (1, 2)			1 (1, 2)			9.93 × 10^−6^		
**Frequency of taking a walk**										
No walks		58%			37%			5.12 × 10^−5^		
Everyday		15%			30%					
Several times a week		12%			18%					
Once a week		4%			8%					
Less often		11%			8%					
**Church attendance**										
No attendance		53%			38%			1.43 × 10^−5^		
occasionally		33%			48%					
regularly		14%			14%					
**Physical activity**										
Unable to get out of bed		20%			3%			8.19 × 10^−30^		
Able to move inside the house but unable to leave the house		34%			28%					
Leaves the house only when necessary		11%			16%					
Able to go for a walk		30%			45%					
Performs physical exercise		4%			8%					
**Duration of walks**										
does not go for walk		58%			37%			2.56 × 10^−15^		
less than 15 min		4%			2%					
15-30 min		8%			16%					
30-60 min		14%			26%					
more than 1 h		16%			19%					
**Guests and Communication**										
	receiving guests		visiting		going out		communication through phone/Internet		using Internet	
cluster	0	1	0	1	0	1	0	1	0	1
does not	40%	25%	76%	42%	40%	25%	40%	12%	94%	88%
1-2 times a year	16%	18%	14%	16%	12%	8%	2%	3%	2%	2%
1-2 times a month	27%	40%	8%	29%	15%	13%	11%	16%	2%	2%
1-2 times a week	15%	14%	2%	12%	17%	23%	21%	32%	2%	2%
everyday	2%	3%	0%	1%	16%	31%	26%	37%	0%	6%
*p*-value	1.09 × 10^−7^		1.35 × 10^−7^		5.11 × 10^−19^		5.35 × 10^−30^		6.35 × 10^−8^	

Note: *—factors used in clusterization. *p*-values calculated using a Mann–Whitney U test for continuous variables or chi-squared test for categorical variables. Continuous variables are represented by median [IQR]. IQR—interquartile range; BMI—body mass index; CVD—cardiovascular diseases; DM2—diabetes mellitus; COPD—chronic obstructive pulmonary disease; AADs—aging-associated diseases; ADL—activity of daily living; IADL—instrumental activity of daily living; GS—geriatric syndrome; GDS—geriatric depression scale; MMSE—mini-mental state examination; SPPB—Short Physical Performance Battery.

**Table 5:** Differences in the factors (only significant) in the least successful aging cluster.

			Cluster 0		Cluster 1. The Least Successful Aging		*p*-Value	
men			289		51		0.02	
women			792		209			
age (median [IQR])			93 (92, 95)		94 (92, 96)			
**Family history**								
Family history of cognitive decline			12%		32%		7.21 × 10^−14^	
**Lifestyle**								
Life-long hobby			46%		29%		4.78 × 10^−6^	
**Education**								
Primary education			6%		16%		1.64 × 10^−8^	
Middle school			15%		14%			
High school			15%		23%			
Vocational education			4%		6%			
High school and vocational education			17%		13%			
Incomplete higher education			2%		2%			
Higher education			38%		25%			
Doctoral degree			3%		1%			
**Job type**								
Intellectually and physically demanding job			22%		35%		3.01 × 10^−4^	
Intellectually demanding job			35%		30%			
Physically demanding job			43%		35%			
Age of retiring			65 (60, 70)		60 (55, 68)		1.27 × 10^−5^	
**Income at the peak of the career**								
Low			9%		18%		7.72 × 10^−5^	
Middle			76%		71%			
High			15%		11%			
**Physical examination**								
Clock-drawing test			7 (5, 9)		3 (0, 5)		3.39 × 10^−41^	
Waist circumference			90 (81, 98)		87.5 (78, 95)		8.88 × 10^−4^	
MMSE *			25 (22, 27)		15 (11, 16)		2.09 × 10^−135^	
Age is not a barrier test			3 (2, 4)		5 (3, 5)		1.66 × 10^−28^	
SPPB *			4 (2, 7)		1 (0, 2)		2.43 × 10^−55^	
Charlson comorbidity index			2 (1, 3)		3 (2, 4)		2.01 × 10^−21^	
GDS-5			1 (0, 3)		3 (1, 4)		4.75 × 10^−26^	
**Vision without glasses**								
Good			20%		15%		4.17 × 10^−5^	
Average			43%		32%			
Bad			37%		53%			
**Syndromes and diseases**								
Frontal lobe disorder *			60%		94%		8.45 × 10^−27^	
Hippocampus dysfunction			19%		67%		1.23 × 10^−50^	
Alzheimer’s disease			9%		49%		5.62 × 10^−44^	
Health self-assessment (10 grade scale)			6 (5, 7)		4 (3, 5)		1.29 × 10^−28^	
Quality of life self-assessment (10 grade scale)			7 (5, 8)		5 (3, 7)		1.29 × 10^−22^	
Number of aging-associated diseases			1 (1, 2)		1 (1, 2)		1.44 × 10^−7^	
**Church attendance**								
No attendance			39%		52%		2.42 × 10^−4^	
occasionally			44%		38%			
regularly			15%		10%			
**Physical activity**								
Unable to get out of bed			5%		27%		4.69 × 10^−21^	
Able to move inside the house but unable to leave the house			32%		38%			
Leaves the house only when necessary			15%		7%			
Able to go for a walk			42%		28%			
Performs physical exercise			6%		0%			
**Duration of walks**								
does not go for walk			58%		68%		2.72 × 10^−14^	
less than 15 min			4%		7%			
15−30 min			8%		7%			
30−60 min			14%		10%			
more than 1 h			16%		8%			
**Guests and Communication**								
	receiving guests		visiting		going out		communication through phone/Internet	
cluster	0	1	0	1	0	1	0	1
does not	28%	50%	49%	76%	27%	48%	15%	43%
1−2 times a year	18%	14%	17%	14%	11%	11%	3%	3%
1−2 times a month	37%	22%	23%	9%	15%	15%	15%	13%
1−2 times a week	14%	13%	10%	1%	22%	17%	30%	24%
everyday	4%	1%	1%	0%	25%	9%	37%	17%
*p*-value	2.08 × 10^−8^		6.21 × 10^−17^		8.50 × 10^−14^		9.03 × 10^−24^	

Note: *—factors used in clusterization. *p*-values calculated with a Mann–Whitney U test *t*-test for continuous variables or chi-squared test for categorical variables. IQR—interquartile range; BMI—body mass index; CVD—cardiovascular diseases; DM2—diabetes mellitus; COPD—chronic obstructive pulmonary disease; AADs—aging-associated diseases; ADL—activity of daily living; IADL—instrumental activity of daily living; GS—geriatric syndrome; GDS—geriatric depression scale; MMSE—mini-mental state examination; SPPB—Short Physical Performance Battery.

## Data Availability

The data presented in this study are available on request from the corresponding author. The data are not publicly available due to ethical restrictions.

## Supplementary Materials

The following supporting information can be downloaded at: https://www.mdpi.com/article/10.3390/ijerph19138178/s1, Table S1: Questionnaries; Table S2: List of factors; Table S3: Associations between GSs and long-term, medium-term and short-term factors; Table S4: Association between GSs, AADs, education and type of work; Table S5: Associations between the presence of aging-associated diseases and risk factors.

## Abbreviations

AADs	aging-associated diseases
ADL	activity of daily-living
BMI	body mass index
CC	correlation coefficient
Cholesterol HDL	cholesterol high-density lipoprotein
COPD	chronic obstructive pulmonary disease
CVD	cardiovascular diseases
DM2	diabetes mellitus type 2
FAB	Frontal Assessment Battery
GDS-5	Geriatric Depression Scale
GSs	geriatric syndromes
IADL	instrumental activity of daily-living
IQR	interquartile range
MHT	menopausal hormone therapy
MMSE	the Mini-Mental State Examination
MNA	Mini Nutritional Assessment
OR	odds ratio
SARC-F	a Simple Questionnaire to Rapidly Diagnose Sarcopenia
SPPB	the Short Physical Performance Battery
T3	triiodothyronine
