# Supplementary material for: Analyzing Successful Aging and Longevity: Risk Factors and Health Promoters in 2020 Older Adults
Source: Int J Environ Res Public Health. 2022 Jul 4;19(13):8178. doi: 10.3390/ijerph19138178 (PMC9266557; doi:10.3390/ijerph19138178)
Supplement: Supplementary file 1 [file ijerph-19-08178-s001.zip › ijerph-1750751-supplementary.pdf]

Supplementary Materials

Table S1 Questionnaries

The Chronic Pain Questionnaire	1. Do you experience pain in everyday life (if not, stop here)?<br>0 - no<br>1 - yes<br>2. Does pain limit your daily life?<br>0 - no<br>1 - yes<br>3. Do you take a pain-relieving medication?<br>0 - no<br>1 – yes, rarely<br>2 - yes, often<br>4. Ask the patient to evaluate the severity of pain at the time of filling out the questionnaire on a scale from 1 to 10 (0 - no pain, 10 - unbearable pain): ⌴⌴ points.
The Anxiety Disorders Questionnaire	1. Are you anxious most of the time?<br>0 - no<br>1 - yes<br>2. Are you worrying too much about the little stuff?<br>0 - no<br>1 - yes<br>3. Do you think of yourself as an anxious person?<br>0 - no<br>1 - yes<br>4. Do you often feel nervous?<br>0 - no<br>1 - yes<br>5. Do you often just have enough thoughts to be alarmed?<br>0 - no<br>1 - yes<br>If the total score was 3 or more, the person was considered anxious. If the total score was less than 3, the person was not considered anxious.
The Risk of falls Questionnaire	1. Have you fallen over the past year?<br>0 - no<br>1 - yes, once<br>2 - yes, twice and more<br>2. Where did you fall?<br>0 – I did not<br>1 - only at home<br>2 - only in the street<br>3 – both at home and in the street<br>3. Have you ever had bone fractures as the results of falling from your own height?

	0 - no<br>1 - yes<br>4. Have you undergone surgical procedures for the resulting bone fractures?<br>0 - no<br>1 - yes<br>5. Did the fracture make you care-dependent?<br>0 - no<br>1 - yes, I was taken care of by a nurse<br>2 - yes, I was taken care of by a family member
The Sensory Deficit Questions	1. Do you have any limitations in your daily life due to loss of vision or hearing?<br>0 - no<br>1 - yes<br>2. Do you wear glasses or contact lenses?<br>0 - no<br>1 - no, but I should<br>2 - yes<br>3. Without glasses, your eyesight is:<br>0 - normal / good<br>1 - not very good<br>2 - bad<br>4. When wearing glasses, your eyesight is:<br>0 - I don't need the glasses<br>1 - normal / good<br>2 - not very good<br>3 - bad<br>5. Is your hearing:<br>0 - good<br>1 - slightly reduced<br>2 - significantly reduced<br>6. Do you use a hearing aid?<br>0 - no, I do not need one<br>1 - no, but would like to/should<br>2 - yes
“Age is not a barrier” screening-test	1. Have you lost 5 kg or more in the past 6 months? Yes/No<br>2. Do you have any limitations in your daily life due to decreased vision or hearing? Yes/No<br>3. Over the past year, have you had a fall-related injury or non-injury falls? Yes/No<br>4. Have you been feeling depressed, sad, or anxious over the past weeks? Yes/No<br>5. Do you have problems with memory, understanding, orientation, or the ability to plan? Yes/No<br>6. Do you suffer from urinary incontinence? Yes/No<br>7. Do you have difficulty moving inside the house or outside (walking up to 100 meters or 1 flight of stairs)? Yes/No<br>The interpretation of the results:<br>For each "Yes" 1 point is awarded. If the total score is:

	• 5 and more — senile asthenia is highly probable;<br>• 3—4 points— senile asthenia is moderately probable;<br>• 0—2 points — senile asthenia is unlikely.

Table S2. List of factors

Long-term factors	● father's age when he died (and the reason if younger than 55)<br>● mother's age when she died (and the reason if younger than 65)<br>● family history of cognitive decline (memory loss
Medium-term factors	● marital status<br>● parental status<br>● total number of children<br>● number of biological children<br>● number of adopted children<br>● education<br>● disability<br>● blood donation<br>● type of menopause<br>● age of menopause onset<br>● number of pregnancies<br>● age of the first childbirth<br>● age of the last childbirth<br>● height<br>● age starting to work<br>● age of retiring<br>● type of job<br>● occupation<br>● income at the peak career<br>● religion<br>● age of taking up smoking<br>● age of quitting smoking<br>● residential area for the last 40 years<br>● previously owning a pet previously (cat or dog)<br>● hobby<br>● life-long physical activity
Short-term factors	● number of family members if living with the family<br>● memory problems<br>● clock-drawing test<br>● frontal lobe disfunction<br>● hippocampus disfunction<br>● weight<br>● BMI<br>● waist circumference

	● systolic pressure<br>● diastolic pressure<br>● heart rate<br>● hypertension<br>● atrial fibrillation<br>● benign tumor<br>● deep vein thrombosis in medical history<br>● pulmonary embolism in medical history tuberculosis<br>● osteoarthritis<br>● hip replacement<br>● rheumatoid arthritis<br>● osteoporosis<br>● gout<br>● Parkinson’s disease<br>● prostate disease<br>● venous ulcer<br>● age-related macular degeneration<br>● gastroesophageal reflux disease<br>● thyroid diseases<br>● mental disorder<br>● skin disorders<br>● restless leg syndrome<br>● essential tremor<br>● sleep apnea<br>● Insomnia Severity Index<br>● aortic stenosis<br>● brachiocephalic artery atherosclerosis<br>● Charlson comorbidity index<br>● self-perceived age<br>● what one finds important/bothering<br>● what one enjoys<br>● what one feels is missing in life<br>● “Age is not a barrier" «Age is not a barrier” screening-test<br>● self-assessment of the quality of life<br>● self-assessment of health<br>● current employment status<br>● receiving guests<br>● going visiting<br>● going out<br>● using the Internet<br>● communicating through phone/Internet<br>● financial circumstances<br>● church attendance<br>● smoking status<br>● cigarette smoke exposure<br>● frequency of alcohol intake<br>● amount of alcohol intake (beverage, quantity, ABV)<br>● six or more alcoholic beverages at one time<br>● physical activity<br>● frequency of walks<br>● duration of walks<br>● physical activity limitation<br>● reasons for physical activity limitation

	• glaucoma<br>• cataract<br>• vision with glasses<br>• vision without glasses<br>• hearing<br>• urinary incontinence while coughing, laughing, sneezing, picking up weights<br>• urinary incontinence on the way to the bathroom<br>• faecal incontinence<br>• MMSE<br>• SPPB<br>• cancer<br>• CVD in anamnesis<br>• diabetes type 2<br>• chronic heart failure<br>• chronic anemia<br>• chronic obstructive pulmonary disease<br>• Alzheimer's disease<br>• number of aging-associated diseases<br>• sleeping pattern<br>• GDS-5<br>• owning pets<br>• hobby

Table S3. Associations between GSs and long-term, medium-term and short-term factors.

Factor	GS	Correlation coefficient	p-value	n
Long-term factors (p-value <0.01)				
father's age when he died	Risk of falls	-0.114	1.95×10^{-05}	1529
	Frontal lobe dysfunction	-0.082	2.99×10^{-03}	1494
Family history of cognitive decline (memory loss)	Incontinence	0.118	8.21×10^{-07}	1798
	Sarcopenia	0.082	6.82×10^{-04}	1588
	Orthostatic hypotension	0.108	1.03×10^{-04}	1238
	Depression	0.192	6.35×10^{-17}	1751
	Risk of malnutrition	0.134	1.04×10^{-07}	1625
	Cognitive impairment	0.168	1.28×10^{-12}	1718
	Frontal lobe disfunction	0.184	3.57×10^{-15}	1739
	Anxiety disorder	0.115	1.09×10^{-04}	1280

	Dependence in ADL	0.079	1.82×10^{-03}	1754
	Polypragmasia	0.093	1.14×10^{-04}	1645
Medium-term factors (p-value<$1.7 \cdot 10^{-3}$)				
age of retiring	Frontal lobe dysfunction	-0.13	4.10×10^{-07}	1680
	Cognitive impairment	-0.141	2.11×10^{-06}	1649
age of menopause onset	Frontal lobe dysfunction	-0.309	5.19×10^{-25}	1095
	Orthostatic hypotension	-0.177	6.13×10^{-07}	791
	Depression	-0.153	1.07×10^{-06}	1096
	Dependence in IADL	0.098	7.73×10^{-04}	1096
age of the first childbirth	Cognitive impairment	-0.111	3.70×10^{-04}	1031
age of quitting smoking	Sensory deficit	0.213	1.57×10^{-03}	205
age of starting to work	Frontal lobe dysfunction	-0.292	3.57×10^{-37}	1716
	Chronic pain	-0.135	1.68×10^{-09}	1763
	Depression	-0.126	1.15×10^{-08}	1723
	Cognitive impairment	-0.091	4.04×10^{-05}	1688
	Risk of falls	-0.088	6.03×10^{-05}	1762
	Anxiety disorder	-0.103	1.29×10^{-04}	1235
	Risk of malnutrition	-0.067	1.70×10^{-03}	1593
disability	Polypragmasia	0.106	3.84×10^{-06}	1768
	Dependence in ADL	0.09	2.35×10^{-05}	1896
	Incontinence	0.083	6.91×10^{-05}	1935
	Risk of malnutrition	0.085	1.64×10^{-04}	1706

	Frailty	0.076	$1.24 X 10^{-03}$	1940
education	Cognitive impairment	-0.228	$1.85 x 10^{-20}$	1854
	Frontal lobe dysfunction	-0.164	$2.70 x 10^{-12}$	1883
	Sarcopenia	-0.085	$1.93 x 10^{-04}$	1692
	Dependence in IADL	-0.091	$4.11 x 10^{-04}$	1912
	Polypragmasia	0.074	$1.11 x 10^{-03}$	1775
previously owning a cat	Chronic pain	0.179	$2.11 x 10^{-11}$	1446
	Sarcopenia	-0.127	$1.10 x 10^{-06}$	1307
previously owning a dog	Chronic pain	0.11	$1.80 x 10^{-05}$	1451
	Sarcopenia	-0.116	$2.01 x 10^{-05}$	1310
	Risk of falls	0.108	$2.86 x 10^{-05}$	1459
	Cognitive impairment	-0.095	$4.40 x 10^{-04}$	1387
hight	Polypragmasia	0.075	$4.24 x 10^{-04}$	1714
hobby	Cognitive impairment	-0.187	$1.29 x 10^{-12}$	1333
	Chronic pain	0.178	$1.87 x 10^{-11}$	1391
	Risk of falls	0.152	$1.62 x 10^{-08}$	1395
	Sarcopenia	-0.15	$2.70 x 10^{-08}$	1266
	Risk of malnutrition	-0.125	$8.18 x 10^{-06}$	1280
income at the peak of career	Cognitive impairment	-0.107	$1.29 x 10^{-03}$	1397
number of pregnancies	Chronic pain	0.114	$5.00 x 10^{-05}$	1274
	Sarcopenia	-0.038	$1.08 x 10^{-03}$	1147
life-long physical activity	Sarcopenia	-0.225	$1.73 x 10^{-16}$	1259

	Chronic pain	0.199	6.79×10^{-16}	1383
	Orthostatic hypotension	0.207	1.83×10^{-10}	960
	Risk of falls	0.151	1.45×10^{-09}	1387
	Dependence in IADL	-0.16	1.29×10^{-08}	1368
	Frontal lobe dysfunction	0.133	3.91×10^{-07}	1341
	Dependence in ADL	-0.141	2.19×10^{-05}	1351
	Risk of malnutrition	-0.134	6.83×10^{-05}	1272
	Sensory deficit	-0.086	1.67×10^{-03}	1386
religion	Frontal lobe dysfunction	-0.136	5.48×10^{-10}	1848
	Depression	-0.102	2.13×10^{-07}	1852
	Orthostatic hypotension	-0.112	2.03×10^{-04}	1305
	Risk of malnutrition	-0.044	1.38×10^{-03}	1716
residential area	Polypragmasia	0.127	1.35×10^{-06}	1422
type of job	Cognitive impairment	-0.176	6.23×10^{-16}	1827
	Dependence in IADL	-0.088	4.76×10^{-05}	1884
	Sarcopenia	-0.091	1.44×10^{-04}	1674
grip strength	Sarcopenia	-0.338	1.12×10^{-46}	1661
	Risk of falls	0.082	5.59×10^{-05}	1787
	Frontal lobe dysfunction	0.084	7.05×10^{-05}	1737
	Chronic pain	0.11	2.22×10^{-07}	1787
	Dependence in IADL	-0.085	1.09×10^{-03}	1762
	Frailty	-0.111	5.89×10^{-05}	1793

Short-term factors (p-value$<5.6 \cdot 10^{-4}$)				
6 or more alcoholic beverages at one time	IADL	-0.127	3.55×10^{-05}	1326
age-related macular degeneration	Frontal lobe dysfunction	-0.129	1.21×10^{-08}	1881
Alzheimer's disease	Dementia	0.325	1.16×10^{-36}	1489
	Frontal lobe dysfunction	0.229	1.30×10^{-18}	1516
	Orthostatic hypotension	0.076	7.57×10^{-07}	1128
	Frailty	0.111	8.92×10^{-05}	1525
	Depression	0.106	2.01×10^{-04}	1507
atrial fibrillation	Polypragmasia	0.195	2.08×10^{-17}	1823
benign tumor	Chronic pain	0.08	1.76×10^{-05}	1924
BMI	Risk of malnutrition	-0.117	1.41×10^{-07}	1765
	Polypragmasia	0.1	2.41×10^{-05}	1701
brachiocephalic artery atherosclerosis	Polypragmasia	0.146	5.40×10^{-10}	1802
	Depression	0.087	3.43×10^{-05}	1895
	Frailty	-0.088	1.82×10^{-04}	1964
	Frontal lobe dysfunction	0.078	3.59×10^{-04}	1882
cataract	Frontal lobe dysfunction	0.148	8.53×10^{-10}	1886
	Chronic pain	0.121	8.75×10^{-08}	1909
	Sarcopenia	-0.095	1.57×10^{-05}	1682
	Risk of falls	0.096	3.90×10^{-05}	1933
	Orthostatic hypotension	0.114	8.21×10^{-05}	1302
	Depression	0.085	4.10×10^{-04}	1893

Charlson comorbidity index	Dementia	0.275	7.31×10^{-34}	1825
	Frontal lobe dysfunction	0.193	6.11×10^{-17}	1844
	Depression	0.186	2.15×10^{-16}	1847
	Polypragmasia	0.185	4.22×10^{-15}	1752
	Frailty	0.116	1.91×10^{-07}	1905
	Urinary or faecal incontinence	0.102	2.31×10^{-06}	1902
	Risk of malnutrition	0.11	3.04×10^{-06}	1700
	Barthel index	0.099	4.25×10^{-06}	1863
	Orthostatic hypotension	0.128	5.07×10^{-06}	1276
	Anxiety disorder	0.125	1.32×10^{-05}	1267
	IADL	0.096	2.65×10^{-05}	1867
	Chronic pain	0.091	4.81×10^{-05}	1848
chronic heart failure	Frontal lobe dysfunction	-0.185	5.22×10^{-16}	1912
	IADL	0.139	4.58×10^{-10}	1956
	Polypragmasia	0.145	5.43×10^{-10}	1824
	Orthostatic hypotension	-0.141	4.22×10^{-07}	1310
	Sarcopenia	0.116	7.94×10^{-07}	1708
	Frailty	0.091	2.13×10^{-05}	1996
	Risk of malnutrition	0.098	2.18×10^{-05}	1757
	Urinary or faecal incontinence	0.084	1.21×10^{-04}	1992
	Barthel index	0.082	1.63×10^{-04}	1946
church attendance	Frontal lobe dysfunction	-0.126	4.73×10^{-09}	1852

	Depression	-0.112	3.14×10^{-08}	1857
	Dementia	-0.094	1.08×10^{-06}	1823
	Chronic pain	0.098	2.63×10^{-04}	1909
clock-drawing test	Dementia	-0.544	3.85×10^{-108}	1508
	Frontal lobe dysfunction	-0.314	7.71×10^{-33}	1503
	Risk of malnutrition	-0.227	7.38×10^{-14}	1366
	Frailty	-0.211	3.44×10^{-12}	1528
	Barthel index	-0.185	4.85×10^{-09}	1496
	Sarcopenia	-0.167	1.01×10^{-08}	1315
	Urinary or faecal incontinence	-0.154	7.59×10^{-07}	1525
	IADL	-0.143	1.33×10^{-06}	1503
	Depression	-0.132	6.25×10^{-05}	1488
	Polypragmasia	0.104	7.12×10^{-05}	1417
communication by phone/Internet	Dementia	-0.272	1.90×10^{-30}	1875
	IADL	-0.147	8.14×10^{-10}	1943
	Frontal lobe dysfunction	-0.131	1.89×10^{-07}	1904
	Risk of malnutrition	-0.136	2.71×10^{-07}	1746
	Frailty	-0.132	3.05×10^{-07}	1975
	Barthel index	-0.117	4.28×10^{-06}	1929
	Polypragmasia	0.105	1.13×10^{-05}	1798
	Sarcopenia	-0.1	1.45×10^{-04}	1698
currently employed	IADL	-0.122	2.57×10^{-07}	1946

	Frailty	-0.097	1.20×10^{-04}	1983
	Barthel index	-0.097	4.47×10^{-04}	1935
CVD in family history	Polypragmasia	0.178	2.96×10^{-14}	1823
	Frontal lobe dysfunction	-0.136	1.43×10^{-08}	1906
	IADL	0.088	3.10×10^{-05}	1952
diabetes type 2	Polypragmasia	0.119	3.38×10^{-07}	1821
Frequency of drinking alcoholic beverages	Sarcopenia	-0.14	1.21×10^{-07}	1324
	IADL	-0.129	7.58×10^{-06}	1439
	Barthel index	-0.137	9.12×10^{-05}	1427
essential tremor	Orthostatic hypotension	0.178	3.02×10^{-10}	1255
	Depression	0.124	6.29×10^{-07}	1792
	Frontal lobe dysfunction	0.098	9.51×10^{-05}	1795
faecal incontinence	Urinary or faecal incontinence	0.398	2.25×10^{-19}	493
	Dementia	0.333	3.81×10^{-13}	459
	Polypragmasia	-0.283	5.22×10^{-08}	378
	Depression	-0.195	1.16×10^{-05}	490
	Barthel index	0.176	2.55×10^{-04}	484
	IADL	0.163	3.84×10^{-04}	480
	Frailty	0.174	4.07×10^{-04}	494
frequency of walks	Risk of malnutrition	-0.148	9.42×10^{-10}	1757
	Dementia	-0.129	3.93×10^{-08}	1883
	Frailty	-0.104	4.90×10^{-06}	1985

frontal lobe disorder	Frontal lobe dysfunction	0.859	0.00 x 10+00	1803
	Dementia	0.386	1.41 x 10^{-59}	1745
	Depression	0.291	1.90 x 10^{-33}	1760
	Orthostatic hypotension	0.216	2.11 x 10^{-14}	1253
	Frailty	0.154	3.11 x 10^{-09}	1802
	Risk of malnutrition	0.145	4.91 x 10^{-08}	1636
	Anxiety disorder	0.14	1.29 x 10^{-06}	1239
	Chronic pain	0.099	6.30 x 10^{-05}	1795
	Barthel index	0.093	4.49 x 10^{-04}	1760
gastroesophageal reflux disease	Dementia	-0.099	1.31 x 10^{-05}	1873
GDS-5	Depression	0.851	0.00 x 10+00	1840
	Anxiety disorder	0.385	6.15 x 10^{-45}	1309
	Frontal lobe dysfunction	0.306	1.88 x 10^{-37}	1773
	Risk of malnutrition	0.312	6.50 x 10^{-35}	1631
	Dementia	0.224	9.08 x 10^{-19}	1746
	Chronic pain	0.203	2.11 x 10^{-16}	1783
	Barthel index	0.193	2.88 x 10^{-14}	1794
	Orthostatic hypotension	0.183	4.02 x 10^{-11}	1243
	Risk of falls	0.148	6.18 x 10^{-09}	1802
	Urinary or faecal incontinence	0.144	1.30 x 10^{-08}	1832
	Frailty	0.124	4.09 x 10^{-06}	1837
	IADL	0.101	8.62 x 10^{-05}	1802

going out	Risk of malnutrition	-0.333	1.95×10^{-40}	1745
	IADL	-0.263	6.20×10^{-30}	1935
	Barthel index	-0.251	3.13×10^{-23}	1922
	Frailty	-0.226	4.41×10^{-20}	1966
	Dementia	-0.223	1.09×10^{-18}	1866
	Urinary or faecal incontinence	-0.178	4.74×10^{-12}	1965
	Depression	-0.168	2.00×10^{-11}	1900
	Sarcopenia	-0.148	5.22×10^{-10}	1696
	Frontal lobe dysfunction	-0.119	2.96×10^{-06}	1893
receiving guests	Depression	-0.15	4.75×10^{-11}	1851
	Frontal lobe dysfunction	-0.138	1.89×10^{-09}	1842
self-assessment of health	Depression	-0.393	5.22×10^{-61}	1684
	Frontal lobe dysfunction	-0.312	9.53×10^{-38}	1675
	Frailty	-0.22	1.61×10^{-18}	1711
	Dementia	-0.198	2.29×10^{-14}	1637
	Barthel index	-0.194	4.01×10^{-14}	1674
	Risk of malnutrition	-0.198	9.67×10^{-14}	1571
	Orthostatic hypotension	-0.192	7.14×10^{-12}	1238
	Risk of falls	-0.163	1.22×10^{-10}	1711
	Chronic pain	-0.16	1.61×10^{-10}	1708
	Urinary or faecal incontinence	-0.124	2.48×10^{-06}	1709
	IADL	-0.098	1.68×10^{-04}	1680

hearing	Sensory deficit	0.423	4.52×10^{-84}	1962
	Chronic pain	0.118	8.01×10^{-08}	1947
	Barthel index	0.116	1.06×10^{-07}	1912
	IADL	0.118	4.99×10^{-07}	1922
	Urinary or faecal incontinence	0.101	2.61×10^{-06}	1957
	Frailty	0.094	1.65×10^{-04}	1959
	Anxiety disorder	0.098	2.05×10^{-04}	1324
	Risk of falls	0.083	3.57×10^{-04}	1952
hippocampus dysfunction	Dementia	0.373	1.85×10^{-51}	1604
	Frontal lobe dysfunction	0.327	2.43×10^{-40}	1647
	Orthostatic hypotension	0.104	2.45×10^{-12}	1186
	Depression	0.181	9.13×10^{-12}	1626
	Frailty	0.13	2.48×10^{-06}	1650
	Risk of malnutrition	0.112	1.00×10^{-04}	1514
	Barthel index	0.1	4.23×10^{-04}	1610
hobby	Dementia	-0.253	1.27×10^{-20}	1341
	Risk of malnutrition	-0.199	6.68×10^{-13}	1291
	Sarcopenia	-0.189	1.94×10^{-11}	1277
	IADL	-0.144	1.43×10^{-07}	1386
	Depression	-0.108	1.00×10^{-04}	1352
	Frontal lobe dysfunction	-0.101	3.11×10^{-04}	1361
	Barthel index	-0.099	4.51×10^{-04}	1368

hypertension	Frontal lobe dysfunction	-0.135	1.90×10^{-09}	1910
	Polypragmasia	0.083	4.07×10^{-04}	1822
	Frailty	0.077	5.23×10^{-04}	1994
Insomnia severity index	Depression	0.319	1.65×10^{-07}	251
	Sensory deficit	0.239	9.59×10^{-05}	246
duration of walks	Risk of malnutrition	-0.325	8.40×10^{-39}	1754
	IADL	-0.253	7.84×10^{-28}	1942
	Barthel index	-0.248	7.95×10^{-24}	1932
	Frailty	-0.223	4.48×10^{-20}	1978
	Depression	-0.202	5.54×10^{-17}	1907
	Dementia	-0.21	7.48×10^{-17}	1877
	Sarcopenia	-0.172	4.02×10^{-13}	1706
	Urinary or faecal incontinence	-0.172	5.88×10^{-12}	1977
memory problems	Risk of malnutrition	0.14	1.33×10^{-08}	1740
memory problems	Urinary or faecal incontinence	0.128	1.99×10^{-08}	1955
memory problems	Sensory deficit	0.106	2.68×10^{-06}	1946
memory problems	Barthel index	0.1	2.21×10^{-05}	1910
memory problems	IADL	0.094	5.36×10^{-05}	1922
memory problems	Depression	0.086	1.47×10^{-04}	1894
memory problems	Chronic pain	0.086	2.71×10^{-04}	1942
MMSE	Dementia	-0.718	9.99×10^{-294}	1904
MMSE	Frontal lobe dysfunction	-0.358	1.12×10^{-54}	1863

MMSE	Frailty	-0.226	8.33×10^{-20}	1902
MMSE	Risk of malnutrition	-0.232	8.05×10^{-19}	1689
MMSE	Urinary or faecal incontinence	-0.178	7.05×10^{-13}	1899
MMSE	Barthel index	-0.181	3.53×10^{-12}	1857
MMSE	Orthostatic hypotension	-0.169	9.62×10^{-10}	1280
MMSE	IADL	-0.145	4.70×10^{-09}	1871
MMSE	Polypragmasia	0.14	5.47×10^{-09}	1733
MMSE	Sarcopenia	-0.145	2.23×10^{-08}	1642
MMSE	Depression	-0.127	2.09×10^{-06}	1846
MMSE	Chronic pain	0.083	2.78×10^{-05}	1851
number of aging-associated diseases	Dementia	0.167	1.48×10^{-11}	1465
	Polypragmasia	0.124	3.77×10^{-06}	1381
	Risk of malnutrition	0.11	1.04×10^{-05}	1390
	Barthel index	0.104	1.82×10^{-05}	1472
	Depression	0.098	6.24×10^{-05}	1484
osteoarthritis	Frontal lobe dysfunction	-0.202	5.72×10^{-20}	1902
osteoarthritis	Sarcopenia	0.125	1.27×10^{-07}	1700
osteoarthritis	Urinary or faecal incontinence	0.123	1.83×10^{-07}	1981
osteoarthritis	Barthel index	0.124	2.01×10^{-07}	1937
osteoarthritis	Polypragmasia	0.121	2.87×10^{-07}	1819
osteoarthritis	IADL	0.107	5.44×10^{-06}	1946
osteoarthritis	Sensory deficit	0.098	2.31×10^{-05}	1932

osteoarthritis	Chronic pain	0.098	6.80×10^{-05}	1925
osteoarthritis	Dementia	-0.07	5.45×10^{-04}	1873
osteoporosis	Risk of falls	0.183	1.99×10^{-14}	1939
osteoporosis	Chronic pain	0.169	1.96×10^{-12}	1911
osteoporosis	Depression	0.126	5.83×10^{-07}	1899
osteoporosis	Anxiety disorder	0.138	9.97×10^{-06}	1311
physical activity	Risk of malnutrition	-0.324	3.31×10^{-38}	1752
physical activity	IADL	-0.263	7.41×10^{-30}	1943
physical activity	Dementia	-0.267	2.94×10^{-27}	1876
physical activity	Frailty	-0.251	3.75×10^{-25}	1978
physical activity	Barthel index	-0.256	4.00×10^{-25}	1934
physical activity	Depression	-0.225	1.06×10^{-20}	1909
physical activity	Urinary or faecal incontinence	-0.206	4.91×10^{-17}	1977
physical activity	Sarcopenia	-0.166	5.60×10^{-12}	1703
physical activity	Frontal lobe dysfunction	-0.148	3.26×10^{-09}	1905
physical activity limitation	Frailty	0.217	4.98×10^{-20}	1928
physical activity limitation	Barthel index	0.211	1.84×10^{-19}	1884
physical activity limitation	IADL	0.197	4.64×10^{-17}	1896
physical activity limitation	Depression	0.173	1.61×10^{-12}	1863
physical activity limitation	Sensory deficit	0.159	7.34×10^{-12}	1922
physical activity limitation	Urinary or faecal incontinence	0.137	8.99×10^{-09}	1927
physical activity limitation	Chronic pain	0.135	1.48×10^{-08}	1923

physical activity limitation	Frontal lobe dysfunction	0.139	1.56×10^{-08}	1857
physical activity limitation	Risk of malnutrition	0.129	9.23×10^{-07}	1723
physical activity limitation	Depression	0.213	2.72×10^{-05}	418
physical activity limitation	Frailty	0.208	6.43×10^{-05}	421
physical activity limitation	Risk of falls	0.093	1.77×10^{-04}	1927
physical activity limitation	Dementia	0.096	3.39×10^{-04}	1828
physical activity limitation	IADL	0.178	5.48×10^{-04}	412
self-assessment of the quality of life	Depression	-0.288	2.44×10^{-31}	1688
self-assessment of the quality of life	Dementia	-0.216	7.66×10^{-17}	1641
self-assessment of the quality of life	Risk of malnutrition	-0.197	8.49×10^{-14}	1573
self-assessment of the quality of life	Frontal lobe dysfunction	-0.181	1.48×10^{-12}	1679
self-assessment of the quality of life	Frailty	-0.177	5.22×10^{-12}	1715
self-assessment of the quality of life	Barthel index	-0.152	5.68×10^{-09}	1678
self-assessment of the quality of life	Sarcopenia	-0.127	1.15×10^{-06}	1565
self-assessment of the quality of life	Orthostatic hypotension	-0.135	1.97×10^{-06}	1243
self-assessment of the quality of life	IADL	-0.106	4.25×10^{-05}	1684
restless leg syndrome	Chronic pain	0.214	3.90×10^{-19}	1768
restless leg syndrome	Anxiety disorder	0.231	1.45×10^{-16}	1301
restless leg syndrome	Risk of falls	0.157	3.83×10^{-11}	1772
restless leg syndrome	Sarcopenia	-0.136	1.32×10^{-07}	1580
restless leg syndrome	Depression	0.106	8.67×10^{-06}	1722
restless leg syndrome	Urinary or faecal incontinence	0.095	1.46×10^{-04}	1775

rheumatoid arthritis	Polypragmasia	0.083	3.24×10^{-04}	1816
«Age is not a barrier" screening-test	Urinary or faecal incontinence	0.445	1.37×10^{-86}	1871
"Age is not a barrier" screening-test	Barthel index	0.384	4.84×10^{-59}	1833
«Age is not a barrier" screening-test	Depression	0.358	2.31×10^{-52}	1842
«Age is not a barrier" screening-test	Risk of falls	0.307	6.36×10^{-39}	1867
«Age is not a barrier" screening-test	Risk of malnutrition	0.301	6.67×10^{-33}	1704
«Age is not a barrier" screening-test	Frailty	0.261	4.36×10^{-26}	1871
«Age is not a barrier" screening-test	IADL	0.242	4.38×10^{-24}	1841
«Age is not a barrier" screening-test	Dementia	0.242	1.26×10^{-21}	1784
«Age is not a barrier" screening-test	Chronic pain	0.224	1.01×10^{-20}	1868
«Age is not a barrier" screening-test	Anxiety disorder	0.264	1.35×10^{-20}	1300
«Age is not a barrier" screening-test	Sensory deficit	0.214	7.48×10^{-20}	1868
«Age is not a barrier" screening-test	Frontal lobe dysfunction	0.159	3.13×10^{-10}	1814
«Age is not a barrier" screening-test	Sarcopenia	0.092	3.88×10^{-04}	1656
sleeping pattern	Chronic pain	0.316	2.93×10^{-27}	1207
sleeping pattern	Depression	0.316	5.83×10^{-27}	1191
sleeping pattern	Anxiety disorder	0.256	1.83×10^{-16}	1118
sleeping pattern	Risk of falls	0.223	2.81×10^{-13}	1207
sleeping pattern	Urinary or faecal incontinence	0.201	1.04×10^{-10}	1206
sleeping pattern	Frontal lobe dysfunction	0.115	8.43×10^{-10}	1185
sleeping pattern	Barthel index	0.183	3.46×10^{-08}	1189
sleeping pattern	Orthostatic hypotension	0.068	5.31×10^{-05}	882

sleeping pattern	Polypragmasia	0.112	2.65×10^{-04}	1166
SPPB	Frailty	-0.717	6.07×10^{-288}	1889
SPPB	Barthel index	-0.38	4.57×10^{-57}	1843
SPPB	Dementia	-0.37	1.14×10^{-52}	1795
SPPB	Urinary or faecal incontinence	-0.296	4.20×10^{-35}	1886
SPPB	Frontal lobe dysfunction	-0.292	3.34×10^{-34}	1813
SPPB	Depression	-0.278	1.93×10^{-29}	1826
SPPB	IADL	-0.263	2.23×10^{-28}	1855
SPPB	Risk of malnutrition	-0.279	2.75×10^{-26}	1665
SPPB	Chronic pain	-0.177	1.57×10^{-12}	1838
SPPB	Risk of falls	-0.177	4.30×10^{-12}	1862
SPPB	Sarcopenia	-0.166	7.21×10^{-11}	1623
SPPB	Orthostatic hypotension	-0.118	1.19×10^{-05}	1260
SPPB	Anxiety disorder	-0.118	1.46×10^{-04}	1282
thyroid diseases	Polypragmasia	0.151	7.18×10^{-11}	1818
thyroid diseases	Dementia	-0.094	1.02×10^{-05}	1872
thyroid diseases	Frontal lobe dysfunction	-0.099	1.03×10^{-05}	1901
urinary incontinence on the way to the bathroom	Urinary or faecal incontinence	0.859	1.71×10^{-142}	491
	Barthel index	0.38	7.27×10^{-17}	482
	Frailty	0.205	4.86×10^{-05}	492
	Sarcopenia	0.203	1.71×10^{-04}	351
	Urinary or faecal incontinence	0.761	4.54×10^{-92}	494

urinary incontinence while coughing, laughing, sneezing, taking weights	Barthel index	0.336	1.37×10^{-12}	485
	IADL	0.238	2.90×10^{-07}	481
	Sarcopenia	0.25	2.52×10^{-06}	351
	Frailty	0.247	4.44×10^{-06}	495
	Frontal lobe dysfunction	0.2	1.07×10^{-04}	467
use of the Internet	IADL	-0.179	3.02×10^{-14}	1949
use of the Internet	Barthel index	-0.162	2.11×10^{-10}	1935
use of the Internet	Dementia	-0.139	1.22×10^{-07}	1881
use of the Internet	Risk of malnutrition	-0.129	3.94×10^{-06}	1752
use of the Internet	Frontal lobe dysfunction	-0.108	1.89×10^{-05}	1909
use of the Internet	Sarcopenia	-0.097	7.61×10^{-05}	1704
use of the Internet	Sensory deficit	-0.092	1.36×10^{-04}	1944
vision with glasses	Sensory deficit	0.206	2.32×10^{-19}	1950
vision with glasses	Anxiety disorder	0.129	1.61×10^{-06}	1320
vision with glasses	Chronic pain	0.095	2.46×10^{-05}	1937
vision with glasses	Urinary or faecal incontinence	0.08	2.07×10^{-04}	1946
vision with glasses	Depression	0.09	2.24×10^{-04}	1878
vision with glasses	Barthel index	0.075	4.08×10^{-04}	1901
vision without glasses	Sensory deficit	0.409	7.15×10^{-78}	1959
vision without glasses	Barthel index	0.153	2.73×10^{-11}	1909
vision without glasses	Risk of malnutrition	0.161	1.15×10^{-10}	1744
vision without glasses	Sarcopenia	0.146	7.18×10^{-09}	1710

vision without glasses	Urinary or faecal incontinence	0.129	1.38×10^{-08}	1954
vision without glasses	IADL	0.132	3.19×10^{-08}	1919
vision without glasses	Dementia	0.125	8.91×10^{-07}	1853
vision without glasses	Depression	0.117	3.38×10^{-06}	1886
going visiting	Frontal lobe dysfunction	-0.427	4.02×10^{-81}	1894
going visiting	Depression	-0.291	3.01×10^{-36}	1899
going visiting	Dementia	-0.201	2.41×10^{-16}	1864
going visiting	Chronic pain	-0.178	9.62×10^{-15}	1928
going visiting	Orthostatic hypotension	-0.205	1.78×10^{-13}	1308
going visiting	Risk of falls	-0.172	2.91×10^{-13}	1946
going visiting	Anxiety disorder	-0.185	3.00×10^{-11}	1318
going visiting	IADL	-0.115	2.23×10^{-06}	1930
going visiting	Barthel index	-0.113	1.02×10^{-05}	1917
going visiting	Risk of malnutrition	-0.108	3.76×10^{-05}	1742
going visiting	Frailty	-0.108	3.78×10^{-05}	1964
going visiting	Sarcopenia	0.079	2.51×10^{-04}	1697
waist circumference	Chronic pain	0.125	1.41×10^{-08}	1643
waist circumference	Risk of malnutrition	-0.14	2.56×10^{-06}	1538
waist circumference	Sarcopenia	-0.102	1.16×10^{-04}	1518
weight	Polypragmasia	0.12	2.61×10^{-07}	1713
weight	Risk of malnutrition	-0.135	1.74×10^{-05}	1765
weight	Frontal lobe dysfunction	-0.099	5.48×10^{-04}	1808

Table S4. Association between GSs, AADs, education and type of work.

Education				
	**GSs**			**Presence of AADs**
	**Sarcopenia**	**Dependence in IADL**	**Polypragmasia**	
Primary education p-value (OR)	0.01(0.52)	0.48 (0.7)	$3.81 \cdot 10^{-4}$(0.51)	1 (1.14)
Middle school p-value (OR)	0.8 (0.93)	0.67 (1.26)	0.37 (1.16)	0.66 (1.3)
High school p-value (OR)	0.01 (1.85)	0.84 (1.09)	0.03 (1.4)	0.53 (0.74)
Vocational education p-value (OR)	$5.54 \cdot 10^{-4}$(0.52)	$1.13 \cdot 10^{-3}$(0.39)	0.01 (1.42)	1 (1.1)
High school and vocational education p-value (OR)	0.86 (0.94)	0.76 (0.95)	0.19 (1.4)	0.5 (2.58)
Unfinished higher education p-value (OR)	0.2 (0.52)	-*	0.39 (0.64)	0.04 (0.22)
Higher education p-value (OR)	$6.72 \cdot 10^{-4}$ (0.59)	$1.68 \cdot 10^{-3}$(0.45)	0.02 (1.3)	$6.5 \cdot 10^{-3}$ (0.49)
Doctoral degree p-value (OR)	$7.83 \cdot 10^{-4}$(0.32)	$2.97 \cdot 10^{-5}$(0.16)	0.13 (1.53)	0.02 (0.29)
P-value	$1.83 \cdot 10^{-4}$	$4.11 \cdot 10^{-4}$	$1.11 \cdot 10^{-3}$	$5.9 \cdot 10^{-4}$
Cohort size	1692	1912	1775	1945
**Job type**				
	**GSs**			**Presence of AADs**
	**Sarcopenia**	**Cognitive impairment**	**Dependence in IADL**	
Intellectually demanding and physically intensive job p-value (OR)	0.01 (1.41)	0.04 (0.81)	$1.16 \cdot 10^{-4}$ (2.52)	$8.1 \cdot 10^{-3}$ (2.08)
Intellectually demanding p-value (OR)	$3.75 \cdot 10^{-5}$ (0.59)	$3.72 \cdot 10^{-7}$ (0.61)	$1.69 \cdot 10^{-6}$ (0.39)	$2.91 \cdot 10^{-7}$ (0.3)
physically intensive job p-value (OR)	0.04 (1.39)	$3.76 \cdot 10^{-16}$ (2.48)	0.15 (1.44)	$1.16 \cdot 10^{-3}$ (2.88)
P-value	$1.44 \cdot 10^{-4}$	$6.23 \cdot 10^{-16}$	$4.76 \cdot 10^{-5}$	$6.86 \cdot 10^{-7}$
Cohort size	1674	1827	1912	1913

Note: * - No participant had both incomplete college degrees and independence in IADL, therefore a comprehensive analysis was impossible. OR – odds ratio; OR - odds ratio.

Table S5. Associations between the presence of aging-associated diseases and risk factors.

Factor	Correlation coefficient	p-value	n
Long-term factors (p-value <0.01)			
Family history of cognitive decline (memory loss)	0.063	7.53 x 10^{-03}	1796
Medium-term factors (p-value<$1.7 \cdot 10^{-3}$)			
education	-0.077	5.90 x 10^{-04}	1945
type of job	-0.112	6.86 x 10^{-07}	1913
grip strength	-0.137	7.85 x 10^{-09}	1790
disability	0.15	3.38 x 10^{-11}	1936
Short-term factors (p-value<$5.6 \cdot 10^{-4}$)			
Alzheimer's disease	0.11	1.87 x 10^{-05}	1524
brachiocephalic artery atherosclerosis	0.079	4.25 x 10^{-04}	1961
Charlson comorbidity index	0.205	1.40 x 10^{-19}	1905
chronic heart failure	0.095	1.87 x 10^{-05}	1993
chronic obstructive pulmonary disease	0.084	1.59 x 10^{-04}	1981
clock-drawing test	-0.189	1.31 x 10^{-13}	1527
communication by phone/Internet	-0.119	1.72 x 10^{-07}	1972
CVD in family history	0.314	1.95 x 10^{-47}	1988
diabetes type 2	0.08	3.69 x 10^{-04}	1984
frontal lobe disorder	0.268	1.83 x 10^{-30}	1801
GDS-5	0.093	7.99 x 10^{-05}	1835
going out	-0.099	1.02 x 10^{-05}	1963
hippocampus dysfunction	0.141	1.64 x 10^{-08}	1649

hobby	-0.134	5.28 x 10^{-07}	1401
duration of walks	-0.106	2.59 x 10^{-06}	1975
memory problems	0.082	2.62 x 10^{-04}	1953
MMSE	-0.196	7.66 x 10^{-18}	1901
number of aging-associated diseases	0.346	1.74 x 10^{-43}	1500
physical activity	-0.107	2.12 x 10^{-06}	1975
physical activity limitation	0.1	1.30 x 10^{-05}	1925
self-assessment of the quality of life	-0.12	9.21 x 10^{-07}	1712
“Age is not a barrier” screening-test	0.17	1.95 x 10^{-13}	1868
SPPB	-0.168	2.00 x 10^{-13}	1884
use of the Internet	-0.138	9.23 x 10^{-10}	1979
vision without glasses	0.109	2.01 x 10^{-06}	1953
